# Cultural sustainability through social networks: a moderated mediation model exploring the psychological dimensions of cultural dissemination

**DOI:** 10.3389/fpsyg.2024.1514693

**Published:** 2025-01-07

**Authors:** Ruolei Chen, Xujia Chen

**Affiliations:** Guangdong Polytechnic of Science and Trade, Guangzhou, China

**Keywords:** social networks, diversity of cultural interactions, cultural empathy, cultural adaptability, cultural sustainability, psychological flexibility

## Abstract

**Introduction:**

The present study examines the role of social network diversity in fostering cultural sustainability among Chinese social media users.

**Methods:**

Utilizing a quantitative methodological approach, data was gathered from a sample of 1,200 active users across various Chinese social media platforms. Participants completed surveys assessing the diversity of their cultural interactions on these platforms, their levels of cultural empathy, cultural adaptability, and the sustainability of cultural practices.

**Results:**

The findings indicate that greater diversity in social media interactions is significantly associated with higher levels of cultural empathy, which in turn enhances both cultural adaptability and sustainability. Furthermore, psychological flexibility was found to moderate these relationships, suggesting that individuals with higher flexibility are better able to leverage diverse interactions into sustainable cultural practices.

**Discussion:**

These results emphasize the potential of social media as a tool for cultural preservation and adaptation in the face of globalization. Implications for policy makers and social media platforms center on fostering environments that support diverse cultural exchanges to enhance cultural sustainability. This research contributes to the understanding of how digital interactions can influence the long-term maintenance and adaptation of cultural heritage in a rapidly changing world.

## Introduction

The advent of social media has profoundly transformed how cultures interact and influence each other in today’s globalized world (Cheng et al., 2021; Sheldon et al., 2020). As platforms that facilitate instantaneous and borderless communication, social networks have become significant arenas for cultural exchange and interaction (Rampersad and Althiyabi, 2020). Recent studies highlight the pivotal role that social media plays in cultural dissemination (Dilleen et al., 2023; Khan et al., 2021), with statistics indicating that over 50% of internet users utilize these platforms to learn about different cultural practices and viewpoints (Global Web Index, 2021). This growing trend illuminates the potential of social networks to not only spread cultural knowledge but also to foster greater understanding and appreciation among diverse populations.

However, the impact of such interactions on cultural sustainability (Soini and Dessein, 2016)—a critical aspect of maintaining the vibrancy and continuity of cultural identities in the face of global influences—remains less understood. As globalization accelerates cultural exchanges (Ergashev and Farxodjonova, 2020), there is a pressing need to explore how these digital interactions can sustain cultures rather than dilute them. This study aims to fill this gap by examining the psychological dimensions of cultural interactions on social networks and their implications for cultural sustainability. By focusing on the underlying psychological processes, this research seeks to provide insights into how digital platforms might support or hinder the preservation and adaptation of cultural practices over time.

The study addresses threefold objectives, each designed to assess the underlying nexus within the realm of cultural interactions on social networks. The first objective is to explore the direct relationships between diversity of cultural interactions—as facilitated by social media—and various dimensions of cultural dynamics, specifically cultural adaptability (Qtaishat et al., 2020) and sustainability (Soini and Dessein, 2016). This exploration is crucial in understanding how frequent and varied intercultural exchanges impact individuals’ ability to adapt to and sustain diverse cultural practices in a globalized context. By examining these relationships, the study aims to shed light on how digital platforms can serve as effective tools in fostering cultural resilience and continuity amid rapid global changes (Michel-Villarreal et al., 2021). Research indicates a significant gap in understanding these direct effects, with many studies focusing on immediate outcomes like awareness and tolerance (Shyryaeva and Trius, 2013; Verkuyten and Killen, 2021), rather than long-term adaptive and sustainable behaviors. This makes our study particularly timely and relevant, as it seeks to bridge this gap by providing empirical evidence on the enduring impacts of digital cultural interactions. The investigation into how diversity of cultural interactions influences adaptability and sustainability not only contributes to theoretical advancements but also offers practical insights for policy makers and educators aiming to leverage social networks for cultural preservation and enhancement. As the global landscape continues to evolve, understanding these dynamics becomes increasingly important in developing strategies that promote cultural diversity and resilience in the face of ongoing changes (Michel-Villarreal et al., 2021). This research thus stands to make a significant contribution to the discourse on cultural sustainability (Ergashev and Farxodjonova, 2020), offering a deeper understanding of the complex interplay between digital interactions and cultural dynamics.

The second objective delves into the mediating mechanism of cultural empathy. Cultural empathy refers to the ability to understand, appreciate, and interact effectively with people from cultures different from one’s own (Pedersen and Pope, 2010; Jami et al., 2024). It seeks to examine how empathy cultivated through diverse cultural interactions can influence the extent to which individuals adapt to and sustain cultural practices. The mediating role of cultural empathy is vital, as it potentially transforms superficial interactions into deeper understanding and integration of diverse cultural norms (Jiang and Wang, 2018). By identifying this mediating pathway, the study aims to contribute significantly to the literature, addressing a noted research gap in how psychological processes mediate the effects of digital cultural interactions on long-term cultural sustainability. Through these objectives, the research reinforces the need for a more nuanced understanding of the psychological underpinnings that facilitate or hinder cultural adaptability and sustainability in the digital age.

The third objective of this study is to investigate the moderating role of psychological flexibility in the relationship between diversity of cultural interactions and cultural empathy, culminating into improved cultural adaptability and sustainability. Psychological flexibility, which refers to the ability to adapt one’s thoughts and behaviors to new, changing, or challenging situations (Doorley et al., 2020), is hypothesized to enhance the effectiveness of cultural interactions in fostering empathy and sustainability. There is a notable research gap in understanding how individual differences in psychological flexibility affect the processing and integration of cross-cultural experiences (Carreno et al., 2023). By examining this moderating effect, the study aims to contribute to the literature by highlighting how varying levels of psychological flexibility can influence the extent to which individuals benefit from diverse cultural interactions. This could have significant implications for designing more effective multicultural training programs and interventions that take into account individual differences in adaptability and responsiveness to cultural diversity.

Last but not the least, our hypothesized relationships are firmly anchored in cultural intelligence theory (CIT, Ang and Van Dyne, 2015), which provides a comprehensive framework for understanding the interactions among the constructs in our model. This theory elucidates how cognitive, motivational, and behavioral components of cultural intelligence influence an individual’s ability to effectively engage and adapt within diverse cultural contexts (Thomas et al., 2015). CIT supports our hypotheses that diversity of cultural interactions on social networks enhances cultural empathy, which in turn influences cultural adaptability and sustainability. Furthermore, the theory underpins our prediction that psychological flexibility, a key aspect of the behavioral component of cultural intelligence, moderates these relationships. By leveraging CIT (Ang and Van Dyne, 2015), our study not only explores the direct and mediated effects of cultural interactions but also the nuanced ways in which individual capabilities enhance the depth and effectiveness of these interactions. This theoretical grounding ensures that our investigation into cultural dynamics on social networks is both comprehensive and precise, providing valuable insights into the mechanisms that promote cultural understanding and sustainability in a digitally connected world.

### Hypotheses development and conceptual model

Diversity of cultural interactions refers to the extent and variety of an individual’s engagement with different cultures through social networks (Caiazza and Volpe, 2015). It captures both the frequency and breadth of cultural perspectives encountered (Howitt, 2005). Studies, such as Ghermandi et al. (2020) and Zhang et al. (2022), identify this diversity through activities like participating in discussions, sharing content, and interacting with culturally diverse users. Such interactions provide access to a global repository of cultural knowledge and perspectives (Schwarzenthal et al., 2020), broadening horizons and influencing attitudes toward other cultures. This engagement fosters greater cultural awareness and sensitivity, making it pivotal for cross-cultural understanding (Howitt, 2005).

Cultural empathy is the ability to understand and share the feelings of individuals from different cultural backgrounds (Pedersen and Pope, 2010; Jami et al., 2024). It extends beyond basic cultural awareness to establish emotional connections with others (Butovskaya et al., 2021). Empathy facilitates effective communication and appropriate responses to behaviors rooted in different cultural contexts (Drummond, 2022). In global networks, it is essential for fostering smoother, respectful exchanges across boundaries, reducing conflicts, and enhancing collaboration.

The relationship between diversity of cultural interactions and cultural empathy is strongly positive, as supported by cultural intelligence theory (CIT). CIT posits that exposure to varied cultural contexts enhances cognitive and emotional understanding of different cultures (Ang and Van Dyne, 2015). Frequent engagement with diverse cultural content expands knowledge and sensitivity, fostering deeper emotional resonance with people from diverse backgrounds (Afsar et al., 2020). Empirical research confirms that frequent interaction with diverse cultures—digitally or in person—develops stronger cultural empathy (Pedersen and Pope, 2010; Jami et al., 2024). These interactions challenge cultural schemas and encourage inclusive thinking, essential for empathy. Social media’s role in facilitating sustained cultural exchanges further supports this perspective (Lee et al., 2020). By engaging with varied narratives online, individuals gain insights into cultural norms and develop the ability to empathize with others (Kramsch and Uryu, 2020). This understanding is crucial in bridging cultural gaps and fostering inclusivity (Lähdesmäki and Koistinen, 2021).

Thus, diversity of cultural interactions on social networks drives cultural empathy, enriching emotional understanding and societal harmony. By expanding exposure to diverse cultural narratives, social media enhances empathy essential for navigating global complexities.

*H1*: Diversity of cultural interactions on social networks is positively associated with cultural empathy.

Cultural adaptability and sustainability are critical for understanding how cultures evolve and remain relevant in a globalized world. Cultural adaptability refers to the ability of individuals and communities to adjust cultural practices and behaviors to meet new environmental and social conditions (Qtaishat et al., 2020). This adaptability facilitates smooth interactions and mutual respect among diverse groups, helping navigate cultural clashes or integration (Wang and Goh, 2020). Cultural sustainability focuses on preserving and continuing cultural heritage across generations despite globalization’s pressures (Soini and Dessein, 2016). It involves safeguarding cultural practices, languages, and beliefs that define a community’s identity while integrating them into broader societal contexts, promoting a diverse yet unified world (Alivizatou-Barakou et al., 2017; Kong et al., 2021). Together, these concepts emphasize the roles of innovation and preservation in cultural evolution.

Cultural empathy is expected to bridge cultural adaptability and sustainability, enabling more profound and effective interactions across diverse cultural landscapes. Empirical research supports this connection. For example, individuals with high cultural empathy are better equipped to adjust behaviors in multicultural settings, a core aspect of adaptability (Thoresen, 2020). This ability to thrive in diverse settings contributes to the long-term sustainability of cultural practices (Kantabutra and Ketprapakorn, 2021). Additionally, fostering environments that value and integrate multiple cultural perspectives ensures the preservation of unique cultural identities, preventing erosion by dominant cultures (Cundiff et al., 2009).

The theory of cultural intelligence (CIT) underpins these relationships, emphasizing that empathetic skills developed through diverse cultural interactions enhance adaptability and integration into different cultural contexts (Thomas et al., 2015). This adaptability is not just about survival but thriving in new environments while respecting and learning from existing cultural frameworks (Qtaishat et al., 2020). Cultural empathy thus acts as a catalyst for adaptability in diverse settings and for preserving cultural heritage, ensuring practices evolve to enrich the global cultural mosaic.

*H2*: Cultural empathy is positively associated with cultural adaptability.

*H3*: Cultural empathy is positively associated with cultural sustainability.

The mediating role of cultural empathy is pivotal in understanding the transformative effects of diverse cultural interactions on cultural adaptability and sustainability. Nurtured through extensive interactions across cultures, cultural empathy facilitates the understanding and application of cultural knowledge in adaptive behaviors (Drummond, 2022). It enhances the capacity to interpret and respond to cultural nuances, supporting adaptive changes while preserving cultural identity (Pedersen and Pope, 2010; Jami et al., 2024). Empathetic individuals are more likely to adopt sustainable practices that honor cultural heritage while adapting to global influences (Kidd, 2019).

Empirical evidence underscores this mediation. For instance, Kong et al. (2020) demonstrate that cultural intelligence significantly influences intercultural adaptation through empathy in service encounters, enabling effective interactions in diverse settings. Similarly, Zheng et al. (2021) found that empathic concern mediates the relationship between cultural backgrounds and social support-seeking behaviors, illustrating empathy’s role in managing relationships and well-being in multicultural environments.

These findings highlight cultural empathy’s ability to bridge cognitive understanding and emotional resonance, enhancing both personal and communal adaptability in diverse cultural contexts. It acts as a crucial intermediary that links theoretical insights with practical actions, ensuring cultural evolution respects heritage while responding to contemporary demands. Thus, cultural empathy fosters a deeper connection between diverse cultural dynamics and adaptive outcomes, making it integral to sustaining cultural practices in a globalized world.

*H4*: Cultural empathy mediates the relationship between diversity of cultural interactions on social networks and cultural adaptability.

*H5*: Cultural empathy mediates the relationship between diversity of cultural interactions on social networks and cultural sustainability.

Psychological flexibility refers to an individual’s ability to adapt thoughts, emotions, and behaviors to situational demands while aligning with their values and goals (Doorley et al., 2020). Central to psychological theories such as Acceptance and Commitment Therapy (ACT, Hayes et al., 2011), psychological flexibility encompasses cognitive, emotional, and behavioral components. Cognitive flexibility involves shifting perspectives to address challenges effectively (Ionescu, 2012). Emotional flexibility is the capacity to manage emotional responses based on context, maintaining balance (Fu et al., 2020). Behavioral flexibility refers to adjusting actions to align with values despite challenges (Lea et al., 2020).

Research highlights the role of psychological flexibility in mental health and well-being by promoting adaptability and resilience. It enables individuals to cope with stress and remain engaged in their goals while responding to life’s challenges with mindfulness (Doorley et al., 2020; Lea et al., 2020). This flexibility ensures responses that are thoughtful rather than impulsive, fostering long-term benefits.

Psychological flexibility is posited to moderate the relationship between the diversity of cultural interactions and cultural empathy by enhancing the capacity to process and integrate diverse cultural experiences. ACT suggests that flexibility aids individuals in remaining open to new experiences without judgment, which is crucial when navigating diverse cultures (Hayes et al., 2011). This openness fosters nuanced understanding, central to developing cultural empathy. For example, individuals with high psychological flexibility navigate complex cultural situations more effectively, adapting behaviors and perspectives to unfamiliar or conflicting norms (Lea et al., 2020). Such adaptability significantly influences how cultural experiences translate into empathetic understanding.

Additionally, psychological flexibility is vital when cultural interactions are challenging or involve conflicting norms. It helps individuals manage emotional responses and maintain curiosity, preventing defensive or insular attitudes (Fu et al., 2020; Doorley et al., 2020). By moderating the relationship between diverse cultural interactions and cultural empathy, psychological flexibility ensures that individuals derive the maximum benefits from cultural diversity. This promotes deeper empathetic engagement, enhancing intercultural competence and fostering better interpersonal relations and cultural integration.

Thus, psychological flexibility moderates the pathway from diverse cultural interactions to cultural empathy, ensuring individuals leverage psychological traits to enhance cultural understanding and cooperation. This theoretical insight underscores its importance in promoting effective engagement across cultural boundaries.

*H6*: Psychological flexibility moderates the relationship between diversity of cultural interactions on social networks and cultural empathy.

Psychological flexibility moderates the mediated relationships between the diversity of cultural interactions and both cultural adaptability and sustainability through cultural empathy. Individuals with high psychological flexibility are better at adjusting their thoughts, emotions, and behaviors in response to new cultural experiences (Lea et al., 2020). This adaptability enhances the impact of diverse cultural interactions on cultural empathy, enabling better integration of cultural information (Giovannetti et al., 2022). Consequently, their enhanced cultural empathy translates into higher adaptability and sustainability. High psychological flexibility allows individuals to navigate cultural differences without becoming defensive, fostering deeper empathetic connections essential for adapting to and sustaining cultural practices (Ang and Van Dyne, 2015; Sánchez-Millán et al., 2022). Conversely, low psychological flexibility hinders the ability to process and adapt to diverse cultural interactions, weakening cultural empathy and its positive effects on adaptability and sustainability. These individuals may resist behavioral changes required for integrating new cultural insights. Empirical evidence supports this moderated mediation. Dooley et al. (2020) show that psychological flexibility enhances individuals’ capacity to cope with complex environments, while Biglan (2009) finds that higher flexibility improves interpersonal relationships and adaptation to social norms, both critical for cultural adaptability and sustainability.

In summary, psychological flexibility amplifies or diminishes the effects of cultural interactions on adaptability and sustainability through cultural empathy, emphasizing its pivotal role in enhancing intercultural competence.

*H7*: Psychological flexibility moderates the mediated relationship between diversity of cultural interactions on social networks and cultural adaptability via cultural empathy.

*H8*: Psychological flexibility moderates the mediated relationship between diversity of cultural interactions on social networks and cultural sustainability via cultural empathy.

**Figure 1:**
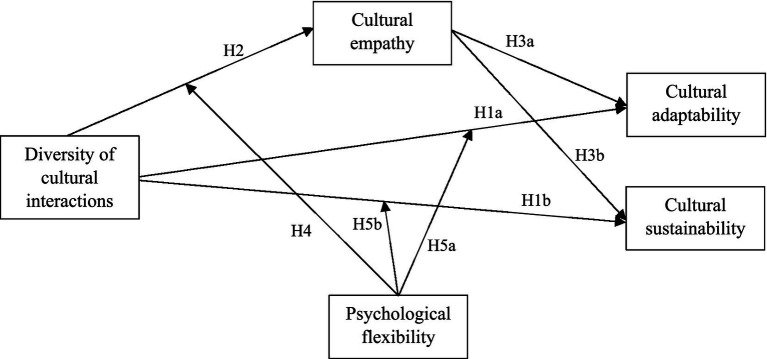
Conceptual model of cultural sustainability.

The conceptual model of the study is presented in Figure 1.

### Research methodology

The methodology for this study on cultural sustainability through social networks in China adopts a quantitative research design to explore how diversity in cultural interactions among social media users influences cultural empathy, adaptability, and sustainability. This design is suitable for testing the hypothesized relationships and effects at scale, enabling the use of statistical analysis to interpret the data collected from the target population.

#### Research design

The research utilizes a cross-sectional survey methodology, which involves collecting data at a single point in time. This approach is efficient for describing the characteristics of a large sample and understanding the relationships between variables in a specified model. The survey consists of standardized instruments to measure the diversity of cultural interactions, cultural empathy, cultural adaptability, and sustainability, as well as psychological flexibility as a moderator.

#### Study population and sampling

The study focuses on Chinese social media users, given the vibrant and diverse nature of social media usage within the country. The sample size was determined to be 1,200 participants to ensure adequate power for statistical analysis. In this study, we employed Partial Least Squares Structural Equation Modeling (PLS-SEM), which is well-suited for complex models and does not impose strict assumptions about data distribution. A commonly accepted guideline for determining sample size in PLS-SEM is the “10-times rule,” which suggests that the minimum sample size should be at least ten times the maximum number of inner or outer model paths pointing at any construct in the model. Given our model’s complexity, a sample size of 1,200 exceeds this minimum requirement, ensuring robust and reliable results.

China’s population is approximately 1.4 billion, with a significant portion engaging in social media. As of early 2024, there were about 1.06 billion social media users in China, accounting for 74.2% of the total population (Digital, 2024). This widespread usage underlines the importance of social media platforms in daily life and cultural interactions.

Stratified random sampling was used to select participants, ensuring representation across different age groups, genders, and regions within China. This technique helps in achieving a sample that is representative of the population, reducing sampling bias and improving the generalizability of the findings (Taherdoost, 2016). Surveys were administered through an online platform, which participants could access using a link sent via email or posted on social media platforms. This method facilitated the efficient distribution and collection of survey data while allowing for a wide geographical reach and convenience for participants. The online administration of the survey also enabled real-time data collection and monitoring of response rates, ensuring a streamlined and effective sampling process.

#### Ethical consent

Prior to participation, all respondents were informed about the purpose of the research, the nature of their involvement, and the use of data for academic purposes only. Consent forms were distributed electronically, which participants had to complete before accessing the survey. These forms assured participants of their anonymity and the confidentiality of their responses. Participants were also informed that they could withdraw from the study at any time without any penalty. Additionally, it stated that results would be reported in aggregate form only, ensuring that individual responses could not be traced back to any participant. This letter also highlighted the academic intent of the research and its potential contribution to understanding cultural sustainability in the digital age. This methodology ensures that the research is conducted ethically and responsibly, respecting the privacy and rights of all participants while providing robust data to address the research questions effectively.

#### Demographic profile

The demographic profile of the participants in the study on cultural sustainability through social networks in China provides a comprehensive overview of the sample’s characteristics. The gender distribution among the participants was fairly balanced, with 48% males and 52% females. Age-wise, the majority of the participants were younger, with 33% falling within the 18–24 year age group, followed by 29% in the 25–34 year range, demonstrating a strong representation of younger social media users. The 35–44 year age bracket was represented by 17% of the participants, while those aged 45–54 made up 12%. The smallest group, those 55 years and above, accounted for 9% of the sample, reflecting lesser but significant engagement among older social media users. This diverse age distribution ensures that the study captures a wide range of social media habits and cultural engagement levels across different life stages.

#### Common method biasness

To effectively address common method bias in our study on cultural sustainability through social networks, we implemented several methodological strategies. We diversified the survey formats and scales to mitigate response patterns and reduce potential biases. Additionally, to counteract social desirability effects, the survey was conducted anonymously, encouraging participants to provide honest responses without the influence of perceived social expectations. Moreover, statistical controls were applied during the analysis phase; Harman’s single-factor test was conducted, revealing that no single factor accounted for the majority of the covariance among the measures. Specifically, the largest factor accounted for only 27.59% of the total variance, indicating that common method bias was unlikely to substantially affect the study’s results (Podsakoff et al., 2003). These combined measures helped in safeguarding the integrity and validity of our findings.

### Measures

In our study, we employed a series of Likert-scale questionnaires adapted from established measures to assess various constructs related to cultural sustainability through social networks. These questionnaires were designed on a five-point scale ranging from 1 (strongly disagree) to 5 (strongly agree), allowing for nuanced responses to each statement. The diversity of cultural interactions scale was adapted from existing studies on cross-cultural engagement and social interaction (Caiazza and Volpe, 2015; Howitt, 2005). A sample item from this scale is “I regularly interact with people from cultures different from my own.” For cultural empathy, we used items derived from research on emotional intelligence and cross-cultural communication (Pedersen and Pope, 2010; Jami et al., 2024; Miao et al., 2020). An example item from this measure is “I can easily understand the feelings of people from different cultural backgrounds. The cultural adaptability scale items were adapted from works on cultural intelligence and adaptability in diverse settings (Qtaishat et al., 2020; Wang and Goh, 2020). A representative item is “I quickly adjust my behavior to be appropriate in different cultural contexts.” Cultural sustainability was measured using items influenced by studies on cultural preservation and the impact of globalization on cultural practices (Alivizatou-Barakou et al., 2017; Ergashev and Farxodjonova, 2020; Soini and Dessein, 2016). One such item from the scale is “I support initiatives that promote the preservation of cultural heritage.” Lastly, the psychological flexibility scale was adapted based on research linking cognitive, emotional, and behavioral flexibility to effective problem-solving and adaptation in varied contexts (Doorley et al., 2020; Fu et al., 2018; Ionescu, 2012; Lea et al., 2020). A typical item from this scale is “I can think about problems from multiple viewpoints.” All questionnaire items were initially developed in English and then translated into Chinese using Brislin’s (1970) model of back-translation to ensure linguistic and conceptual accuracy. This rigorous translation process guarantees that the measures are culturally and contextually appropriate for the Chinese audience, maintaining the integrity and reliability of the data collected.

### Analytical strategy

In our study, we utilized the Partial Least Squares Structural Equation Modeling (PLS-SEM) approach to analyze the data collected from the surveys. This analytical strategy is particularly suitable for exploratory research where the goal is to build and expand theoretical models (Hair et al., 2017). The analysis was divided into two key phases: the measurement model and the structural model. First, the measurement model was assessed to confirm the reliability and validity of the constructs. This involved evaluating the internal consistency of the scales, convergent validity, and discriminant validity using criteria such as Cronbach’s alpha, composite reliability, Average variance extracted (AVE), and the Fornell-Larcker criterion. Ensuring robust measurement models helps in substantiating that the scales adequately represent the latent constructs they are supposed to measure. Subsequently, the structural model was analyzed to examine the hypothesized relationships between the constructs. This phase focused on the path coefficients, the significance of these relationships (using bootstrapping as recommended by Hair et al., 2017), and the overall model fit. The structural model analysis allows us to test the direct and indirect effects within the model, providing insights into the dynamics of cultural sustainability as influenced by social network interactions, mediated by cultural empathy, and moderated by psychological flexibility. This dual-phase approach using PLS-SEM ensures a thorough examination of both the measurement properties of the data and the structural relationships between constructs, thereby providing a comprehensive understanding of the model under investigation.

### Research findings

In Table 1, the assessment of multicollinearity involves analyzing the variance inflation factors (VIF) for the variables: diversity of cultural interactions, cultural empathy, psychological flexibility, cultural adaptability, and cultural sustainability. The results show that the variance inflation factors for these variables are within acceptable limits, indicating that multicollinearity is not a concern for this analysis. This effective handling of multicollinearity ensures that the subsequent regression analysis produces reliable and interpretable results.

**Table 1:** Assessment of multicollinearity.

	DCI	CE	PF	CA	CS
DCI		1.21		1.94	2.29
CE				1.09	1.43
PF		2.13		1.17	1.00
CA					
CS					

Table 2 presents the assessment of reliability and validity of constructs used in the study. The reliability of each construct is robust, as indicated by Cronbach’s alpha (*α*) and composite reliability (CR) scores, all of which are above the generally accepted threshold of 0.7, suggesting that the items within each construct consistently measure the same concept. For diversity of cultural interactions, the alpha and composite reliability scores are 0.889 and 0.921 respectively, with an average variance extracted (AVE) of 0.731, which exceeds the recommended value of 0.5, indicating a good level of convergent validity. The factor loadings of the individual items range from 0.732 to 0.965, all above the acceptable threshold of 0.7, further confirming the construct’s validity. Cultural empathy also shows strong reliability with alpha and composite reliability values of 0.871 and 0.901. However, the AVE is 0.569, which is marginally above the cutoff, suggesting adequate, but not strong, convergent validity. The factor loadings for cultural empathy items vary from 0.767 to 0.872, demonstrating sufficient item reliability. Psychological flexibility exhibits alpha and composite reliability scores of 0.858 and 0.889. The AVE for psychological flexibility is 0.634, indicating satisfactory convergent validity. The factor loadings for this construct are somewhat varied, with one item as low as 0.615 but others well above the 0.7 mark, overall supporting the construct’s reliability. Cultural adaptability’s reliability scores are 0.853 for alpha and 0.885 for CR, with an AVE of 0.667, showing good internal consistency and convergent validity. The factor loadings for cultural adaptability items are strong, mostly above the 0.7 line, confirming the construct’s validity. Finally, cultural sustainability has alpha and CR scores of 0.812 and 0.847, and an AVE of 0.690. All factor loadings for cultural sustainability exceed 0.7, emphasizing robust item reliability and construct validity. Overall, these results confirm that the measurement models are reliable and valid, providing a strong foundation for further structural model analysis.

**Table 2:** Assessment of reliability and validity of constructs.

Items	Constructs	α/CR	AVE	Factor loading
DCI	Diversity of cultural interactions	0.889/0.921	0.731	
DCI1				0.853
DCI2				0.732
DCI3				0.965
DCI4				0.825
DCI5				0.883
CE	Cultural empathy	0.871/0.901	0.569	
CE1				0.850
CE2				0.767
CE3				0.769
CE4				0.856
CE5				0.768
CE6				0.872
CE7				
PF	Psychological flexibility	0.858/0.889	0.634	
PF1				0.813
PF2				0.795
PF3				0.615
PF4				0.776
PF5				0.853
PF6				0.737
PF7				0.864
PF8				0.737
PF9				0.798
PF10				0.862
PF11				0.871
CA	Cultural adaptability	0.853/0.885	0.667	
CA1				0.844
CA2				0.793
CA3				0.738
CA4				0.704
CA5				0.898
CA6				0.904
CS	Cultural sustainability	0.812/0.847	0.690	
CS1				0.831
CS2				0.869
CS3				0.773
CS4				0.886
CS5				0.830
CS6				0.799
CS7				0.818

Tables 3, 4 provide critical assessments regarding the discriminant validity of the constructs in the study. Table 3 utilizes the Fornell-Larcker criterion, which compares the square root of the average variance extracted (AVE) for each construct with the correlations among constructs. According to Fornell and Larcker (1981), for adequate discriminant validity, the square root of the AVE of each construct should be greater than the correlations between that construct and any other construct. In this case, the diagonal elements (which represent the square root of AVEs) are all greater than the off-diagonal elements in their respective rows and columns. Table 4 assesses the discriminant validity using the heterotrait-monotrait ratio (HTMT) of correlations, a more recent and stringent criterion suggested by Henseler et al. (2015). An HTMT value less than 0.85 is typically indicative of adequate discriminant validity. The HTMT ratios reported, such as 0.463 between diversity of cultural interactions and cultural empathy and 0.602 between cultural empathy and psychological flexibility, are all well below the 0.85 threshold, further supporting the discriminant validity of the constructs.

**Table 3:** Assessment of Fornell-Larcker.

	DCI	CE	PF	CA	CS
DCI	0.854				
CE	0.521	0.754			
PF	0.446	0.587	0.796		
CA	0.555	0.412	0.409	0.816	
CS	0.663	0.462	0.310	0.363	0.831

**Table 4:** Assessment of HTMT ratio.

	DCI	CE	PF	CA	CS
DCI					
CE	0.463				
PF	0.722	0.602			
CA	0.578	0.518	0.511		
CS	0.490	0.471	0.691	0.367	

Table 5 presents the assessment of the structural model, detailing the relationships within the model, along with their path values, significance levels, *t*-values, R-squared values, effect sizes (*F*^2^), and variance accounted for (VAF) where applicable. This comprehensive analysis helps in understanding the direct, total, indirect, and moderated effects of the constructs involved in the study.

**Table 5:** Assessment of structural model.

	Relationship	Path value	*p* value	*T* value	*R*^2^ value	*F*^2^ value	VAF
Direct effects	DCI➔CE (H1)	0.391	0.000	7.592	0.459	0.228	
	CE➔CA (H2)	0.398	0.000	6.620	0.498	0.183	
	CE➔CS (H3)	0.344	0.000	8.845	0.551	0.222	
Total and indirect effects	DCI➔CA (total effect)	0.565	0.000	8.218			
	DCI➔CS (total effect)	0.547	0.000	6.548			
	DCI➔CE➔CA (H4)	0.312	0.006	3.660		0.216	0.552
	DCI➔CE➔CS (H5)	0.321	0.003	4.678		0.295	0.586
Moderation effects	Moderation of PF on the relationship of DCI➔CE (H6)	0.298	0.000	7.617		0.124	
	Moderation of PF on the relationship of DCI➔CE➔CA (H7)	0.352	0.000	6.371		0.212	
	Moderation of PF on the relationship of DCI➔CE➔CS (H8)	0.301	0.000	8.989		0.214	

VAF, variance accounted for (indirect effect/total effect).

#### Direct effects

The path from diversity of cultural interactions to cultural empathy (H1) is significant, with a path coefficient of 0.391, a *p*-value of 0.000, and a T-value of 7.592. This relationship explains 45.9% of the variance in cultural empathy, indicating a strong effect. Similarly, cultural empathy’s influence on cultural adaptability (H2) and cultural sustainability (H3) are also significant, with path coefficients of 0.398 and 0.344, respectively. Both paths are statistically significant, and the R-squared values indicate that cultural empathy explains 49.8 and 55.1% of the variance in cultural adaptability and sustainability, respectively.

#### Total and indirect effects

The total effects of diversity of cultural interactions on cultural adaptability and cultural sustainability are 0.565 and 0.547, respectively, both significant at the 0.000 level. This suggests strong overall influences when mediated by cultural empathy. The specific indirect effects through cultural empathy (H4 and H5) on cultural adaptability and sustainability have path coefficients of 0.312 and 0.321, respectively, and are significant. The VAF values of 0.552 and 0.586 indicate that a substantial portion of the total effect of diversity of cultural interactions on these outcomes is mediated by cultural empathy.

#### Moderation effects

**Figure 2:**
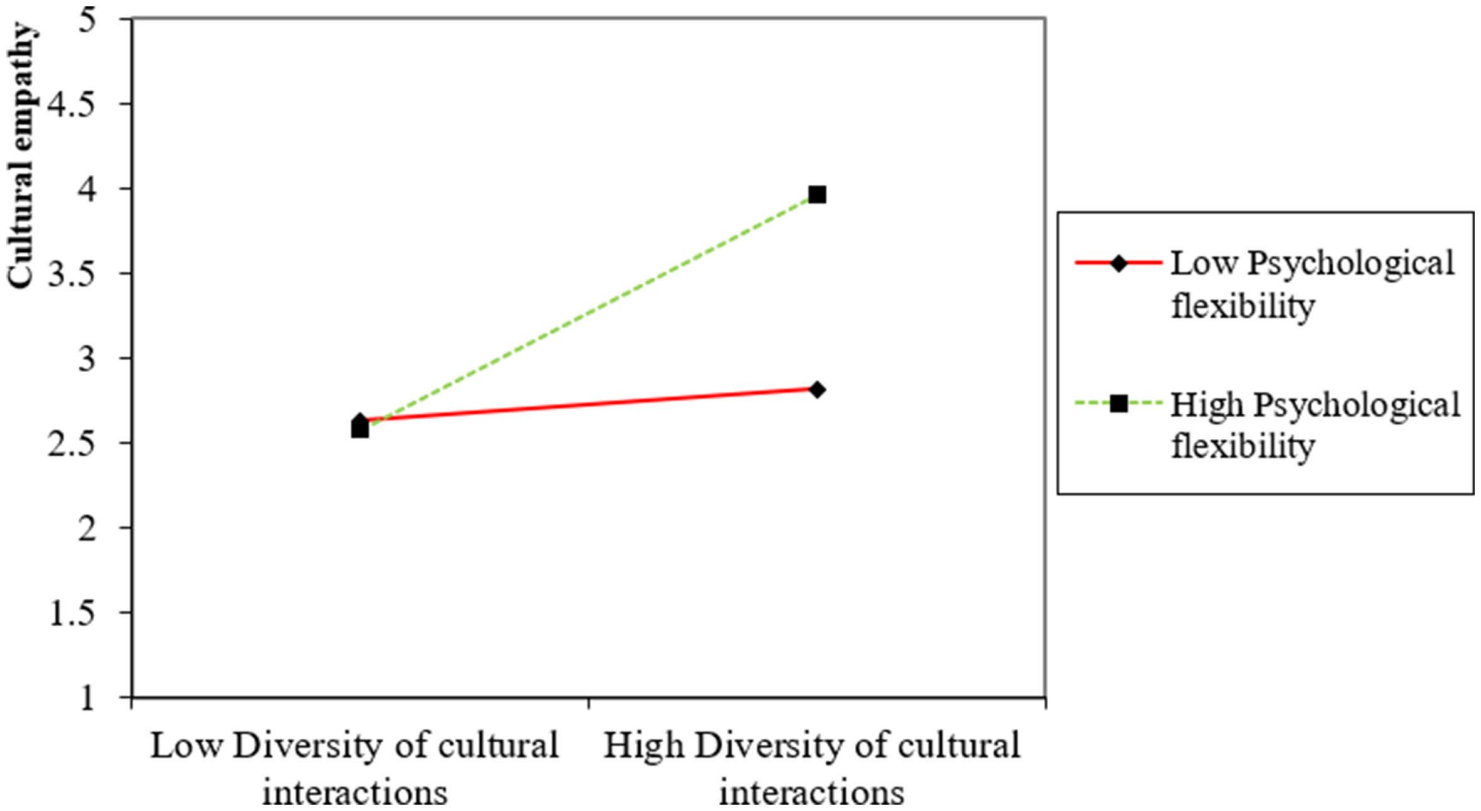
Moderation effect of psychological flexibility on cultural empath.

**Figure 3:**
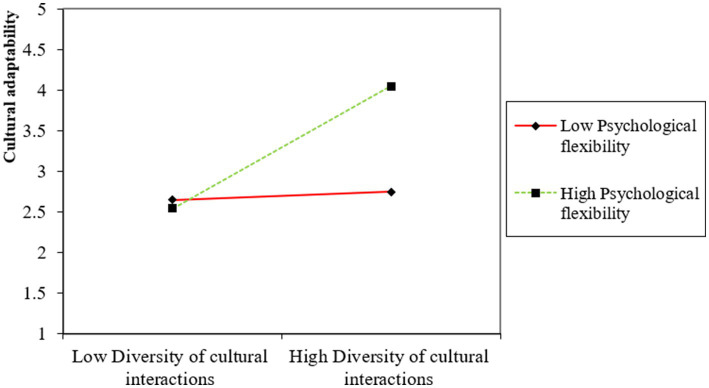
Moderated mediation effect of psychological flexibility on cultural adaptability.

**Figure 4:**
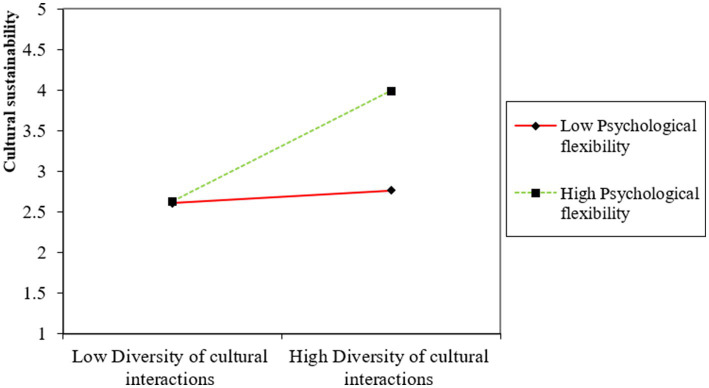
Moderated mediation effect of psychological flexibility on cultural sustainability.

The moderation of psychological flexibility on the direct relationship between diversity of cultural interactions and cultural empathy (H6) shows a path value of 0.298, indicating a significant moderating effect with a notable effect size of 0.124. In addition, psychological flexibility also significantly moderates the indirect effects of diversity of cultural interactions on cultural adaptability (H7) and cultural sustainability (H8) through cultural empathy, with path coefficients of 0.352 and 0.301, respectively. The effect sizes of 0.212 and 0.214 further demonstrate the strength of these moderated mediation effects. The moderation and moderated mediation results are also illustrated in Figures 2–4, which show the variation impacts on cultural empathy and cultural adaptability and sustainability at various levels of psychological flexibility. The analysis confirms that when psychological flexibility is higher, the direct and indirect relationships are more strongly pronounced as compared to when psychological flexibility is lower. Overall, the analysis of the structural model in Table 5 confirms the hypothesized relationships within the study, illustrating the critical roles played by cultural empathy and psychological flexibility in mediating and moderating the effects of diversity of cultural interactions on cultural outcomes. These findings provide strong empirical support for the theoretical framework posited in the study, highlighting the complex interplay of cultural interactions, empathy, and psychological flexibility in promoting cultural adaptability and sustainability.

## Discussion

The study embarked on a comprehensive exploration of how diversity of cultural interactions influences cultural dynamics through the mediating role of cultural empathy and the moderating role of psychological flexibility. The objectives were robustly met, revealing significant insights into the relationships between these variables.

The first objective examined the direct relationship between diversity of cultural interactions and cultural empathy. We found a strong positive relationship, suggesting that individuals engaging in diverse cultural exchanges are more likely to develop empathetic understanding toward other cultures. This finding aligns with Lin et al. (2012), who identified that cultural intelligence enhances intercultural adaptation via empathy. Similarly, Setti et al. (2022) demonstrated that empathy plays a mediating role in how individuals from different cultural backgrounds seek social support, reinforcing the notion that varied cultural interactions enhance empathetic skills. Another study by Hu et al. (2020) also supports this, showing that exposure to diverse cultures significantly boosts cultural empathy, emphasizing the transformative power of social media as a platform for cultural learning and interaction.

The second objectives delved into how cultural empathy mediates the impact of diversity of cultural interactions on cultural adaptability and sustainability. Our findings indicated that cultural empathy significantly fosters both adaptability and sustainability, serving as a critical pathway through which cultural interactions translate into more concrete outcomes. This mediation is crucial, as shown by Blakemore and Agllias (2020), who found that empathy directly influences intercultural communication competence. Similarly, Vinayak and Judge (2018) highlighted that empathy enhances individuals’ ability to adapt and thrive in diverse environments. Furthermore, research by Brown et al. (2019) elucidates how empathy facilitates not just adaptation but also the long-term sustainability of cultural practices.

Moreover, the moderating role of psychological flexibility was found to significantly influence both the direct and mediated relationships within the model. The study found that individuals with high psychological flexibility were better able to leverage their empathetic understandings for greater adaptability and sustainability. This moderation effect aligns with the findings of Cheng et al. (2014), who noted that psychological flexibility enhances social and psychological coping in varied cultural settings. Likewise, Bi and Li (2021) reported that flexibility aids in adjusting to new cultural norms, while research by Esen-Aygun (2018) further corroborates the idea that cognitive flexibility is crucial in maintaining and enhancing interpersonal relationships across different cultures.

### Theoretical implications

The theoretical implications of this study are profound, enhancing our understanding of the interplay between cultural interactions, empathy, and psychological flexibility within the context of globalized social networks. By elucidating these dynamics, the research contributes significantly to several key areas of theoretical discourse.

Firstly, this study enriches the CIT by empirically validating the theory’s propositions regarding the role of cultural interactions in developing cultural empathy. The study demonstrates that diverse cultural interactions, facilitated through social networks (Zhang et al., 2022), not only increase cultural knowledge but also enhance the empathetic abilities necessary for effective cross-cultural engagement. This finding provides a nuanced understanding of how cognitive and emotional components of cultural intelligence are activated and developed through digital platforms (Zhou and Charoensukmongkol, 2022), expanding the theory’s applicability beyond face-to-face interactions to include virtual environments.

Secondly, the study extends the application of the Contact Hypothesis (Paluck et al., 2019) in digital contexts by showing that online cultural interactions can reduce prejudices and enhance understanding, much like direct interpersonal contacts. By demonstrating the mediating role of cultural empathy in this process, the research bridges the gap between abstract cultural exchanges and tangible cultural adaptation and sustainability. This mediational insight suggests that the Contact Hypothesis may be a useful framework for examining the impacts of digital communication on intercultural relations, encouraging further theoretical development in this direction.

Additionally, the examination of psychological flexibility as a moderator in the relationships between cultural interactions, empathy, and cultural outcomes introduces a new dimension to the study of intercultural communication and adaptation. This aspect of the research highlights the importance of individual differences in cognitive and behavioral flexibility, suggesting that the effects of cultural interactions are not uniform but vary according to individual psychological traits. This finding urges theorists to consider psychological flexibility as a critical factor in models of cultural adaptation and sustainability, potentially leading to more personalized approaches in intercultural training and education programs.

Furthermore, this study’s findings advocate for a more integrated approach to understanding cultural dynamics, where emotional and cognitive components are considered as interconnected rather than isolated factors. This holistic view can facilitate more comprehensive theories that address the complexities of cultural interactions in an increasingly interconnected world.

Overall, the theoretical contributions of this study are substantial, offering fresh perspectives and deepening existing theories within the fields of cultural intelligence, intercultural communication, and psychological flexibility. These insights not only advance academic discourse but also provide a solid foundation for future research to build upon, potentially leading to richer, more effective models of understanding and managing cultural diversity in globalized societies.

### Practical implications

The practical implications of this study are significant, offering actionable insights for various sectors including education, corporate settings, policy making, and community development, particularly in enhancing cultural competence and promoting diversity and inclusion.

1. Education and Training: Educators and trainers can integrate the findings of this study into curricula and professional development programs to enhance cultural empathy and adaptability among students and employees. Schools and universities can develop exchange programs and collaborative online international learning (COIL, Borger, 2022) that promote diverse cultural interactions, leveraging social media and digital platforms as tools for global education. Further, training programs can also emphasize the development of psychological flexibility, teaching skills that help individuals adapt to and thrive in diverse environments.
2. Corporate and Organizational Development: Organizations can use these insights to improve their diversity and inclusion strategies. Recognizing the role of cultural empathy in facilitating adaptability and sustainability within diverse work environments, companies can create initiatives that foster diverse cultural interactions, such as international team projects or culturally diverse workgroups. Additionally, human resources departments can incorporate assessments and workshops that focus on enhancing psychological flexibility, aiming to build a more adaptable and culturally competent workforce.
3. Policy Making: Policymakers can apply the findings to promote cultural sustainability and integration within communities. By understanding the mechanisms through which social networks influence cultural dynamics, policies can be designed to support the creation and maintenance of digital platforms that foster positive cultural exchanges. This could include funding for digital literacy programs that teach effective and empathetic communication across cultures, or supporting initiatives that use social media to bridge cultural divides and enhance community engagement.
4. Community Development and Social Work: Community leaders and social workers can use these insights to design interventions that enhance intercultural understanding and cooperation. Programs that encourage community members to engage in social media exchanges with people from different backgrounds can be developed to reduce prejudices and strengthen community ties. Additionally, workshops that train individuals in psychological flexibility can help community members better handle the challenges associated with cultural integration.
5. Digital and Social Media Platforms: The study’s findings can inform the design and moderation of social media platforms to encourage more meaningful and empathetic intercultural interactions. Platform designers can create features that promote exposure to diverse cultural content in a way that fosters understanding and empathy, rather than conflict. Moderation policies could also be developed to support respectful and constructive cultural exchanges, helping to create online environments that are conducive to cultural learning and empathy.

In summary, the practical applications of this research are broad and impactful, offering guidance for enhancing cultural adaptability and sustainability through strategic use of digital interactions and fostering psychological flexibility across various domains of society.

### Limitations and avenues for future studies

Despite the contributions of this study, there are several limitations that need to be acknowledged and could serve as avenues for future research. One of the primary limitations is the reliance on self-reported data, which can introduce biases such as social desirability or response bias. Future studies might incorporate more objective measures of cultural interaction and empathy, such as observational data or third-party assessments, to provide a more comprehensive understanding of these phenomena. Additionally, while the study utilized a cross-sectional design, which provides a snapshot in time, it does not capture the dynamics of how cultural interactions and empathy evolve over time. Longitudinal studies could be valuable in understanding the long-term effects of diverse cultural interactions on empathy and adaptability.

Another limitation is the study’s focus on a specific cultural or demographic context, which may not generalize across different populations or cultural settings. Future research could replicate this study in various cultural contexts to examine the universality of the findings. Moreover, exploring the impact of different types of social media platforms might also yield insights, as different platforms may facilitate different types of interactions and thus may influence cultural empathy and adaptability in diverse ways. Finally, integrating qualitative methods could enrich the quantitative findings, providing deeper insights into the personal experiences and nuances of cultural interactions that are not captured through survey data alone. These approaches would significantly expand the understanding of the complex interplay between cultural interactions, empathy, and psychological flexibility.

## Conclusion

This study significantly contributes to the understanding of how diversity of cultural interactions on social media platforms can enhance cultural empathy, which in turn fosters cultural adaptability and sustainability. By integrating the roles of cultural empathy and psychological flexibility, the findings elucidate the mechanisms through which digital interactions not only enhance personal cultural competence but also contribute to the broader societal goals of cultural preservation and integration. The research highlights the potential of social media as a powerful tool for promoting understanding and cooperation across cultures, highlighting the importance of fostering psychological flexibility to maximize these benefits. As societies continue to direct the complexities of a globalized world, the insights provided by this study offer valuable guidance for leveraging digital platforms to cultivate a more inclusive and culturally aware global community.

## Data Availability

The raw data supporting the conclusions of this article will be made available by the authors, without undue reservation.
